# Case Report: Left ventricular pseudoaneurysm presenting with ventricular tachycardia

**DOI:** 10.3389/fcvm.2026.1903760

**Published:** 2026-09-03

**Authors:** Esra Polat, Mahmut Arslan, Yusuf Çorbacıoğlu, Ahmet Khalousi, Rauf Önder, Candan Güngör

**Affiliations:** 1 Department of Cardiology, Gaziantep City Hospital, Gaziantep, Türkiye; 2 Department of Cardiology, Kilis Prof. Dr. Alaeddin Yavaşca State Hospital, Kilis, Türkiye; 3 Department of Cardiovascular Surgery, Gaziantep City Hospital, Gaziantep, Türkiye; 4 Department of Radiology, Gaziantep City Hospital, Gaziantep, Türkiye

**Keywords:** cardiac magnetic resonance imaging, myocardial infarction, surgical repair, ventricular pseudoaneurysm, ventricular tachycardia

## Abstract

Left ventricular pseudoaneurysm is a rare and life-threatening disorder that can present with a wide range of clinical manifestations, from an asymptomatic disease to sudden cardiac death. This case report presents a 38-year-old patient with no history of cardiovascular disease who presented with ventricular tachycardia and was diagnosed with left ventricular pseudoaneurysm following multiple cardiac imaging investigations. Given the high risk of rupture, early diagnosis of this rare condition is of critical importance, as early surgical intervention can be lifesaving.

## Introductıon

Ventricular tachycardia may be caused by ischemia, acute coronary syndrome, structural heart disease, myocarditis, channelopathies, infiltrative heart diseases, electrolyte imbalances, or genetic syndromes (1, 2). In treatment strategies, identifying the etiology is crucial for medical therapy, implantable defibrillator therapy, or ablation therapy (3). In this case report, we present a young patient who presented with ventricular tachycardia and was found to have a left ventricular pseudoaneurysm.

## Case report

**Figure 1:**
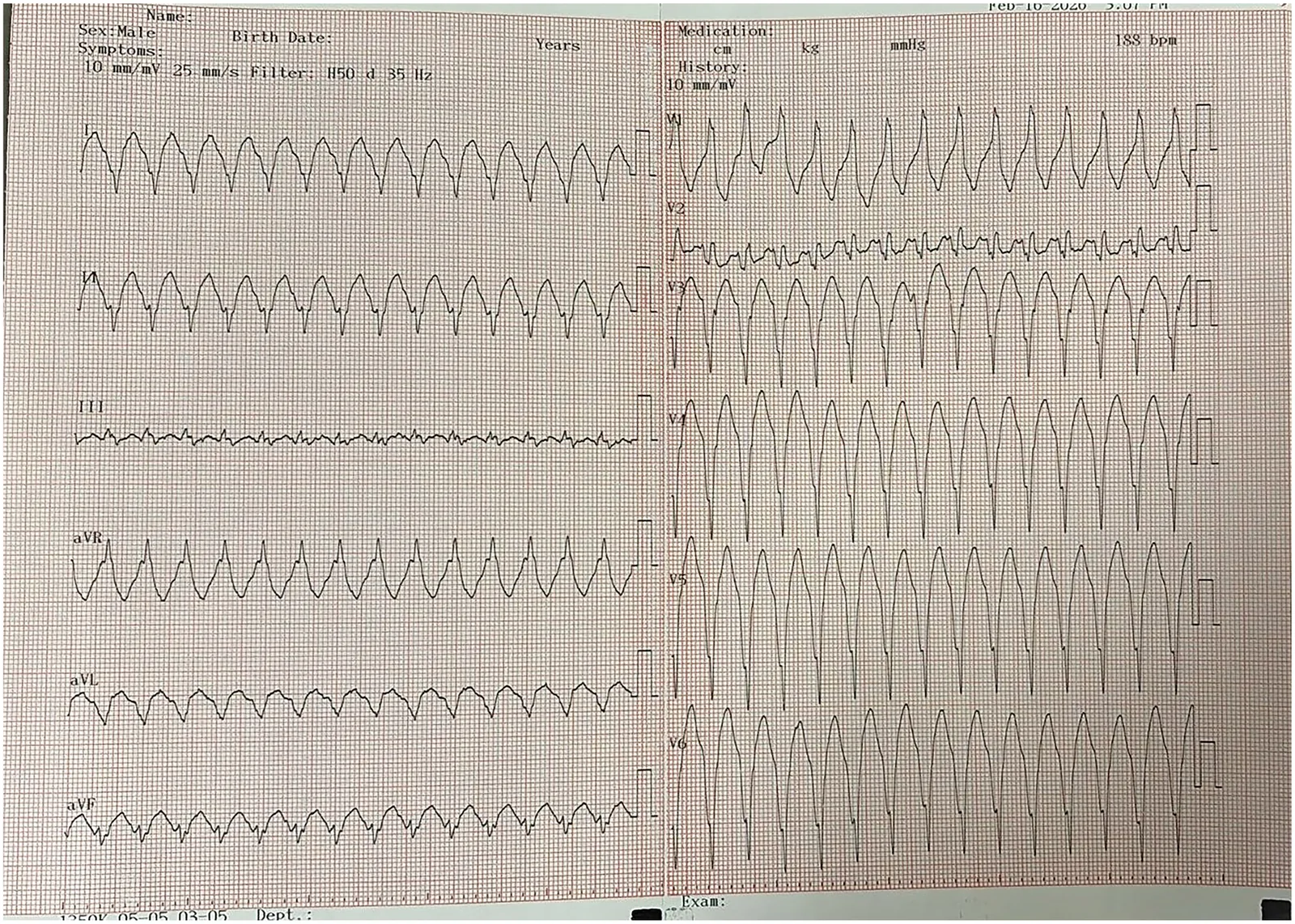
The patient's ECG showing ventricular tachycardia.

**Figure 2:**
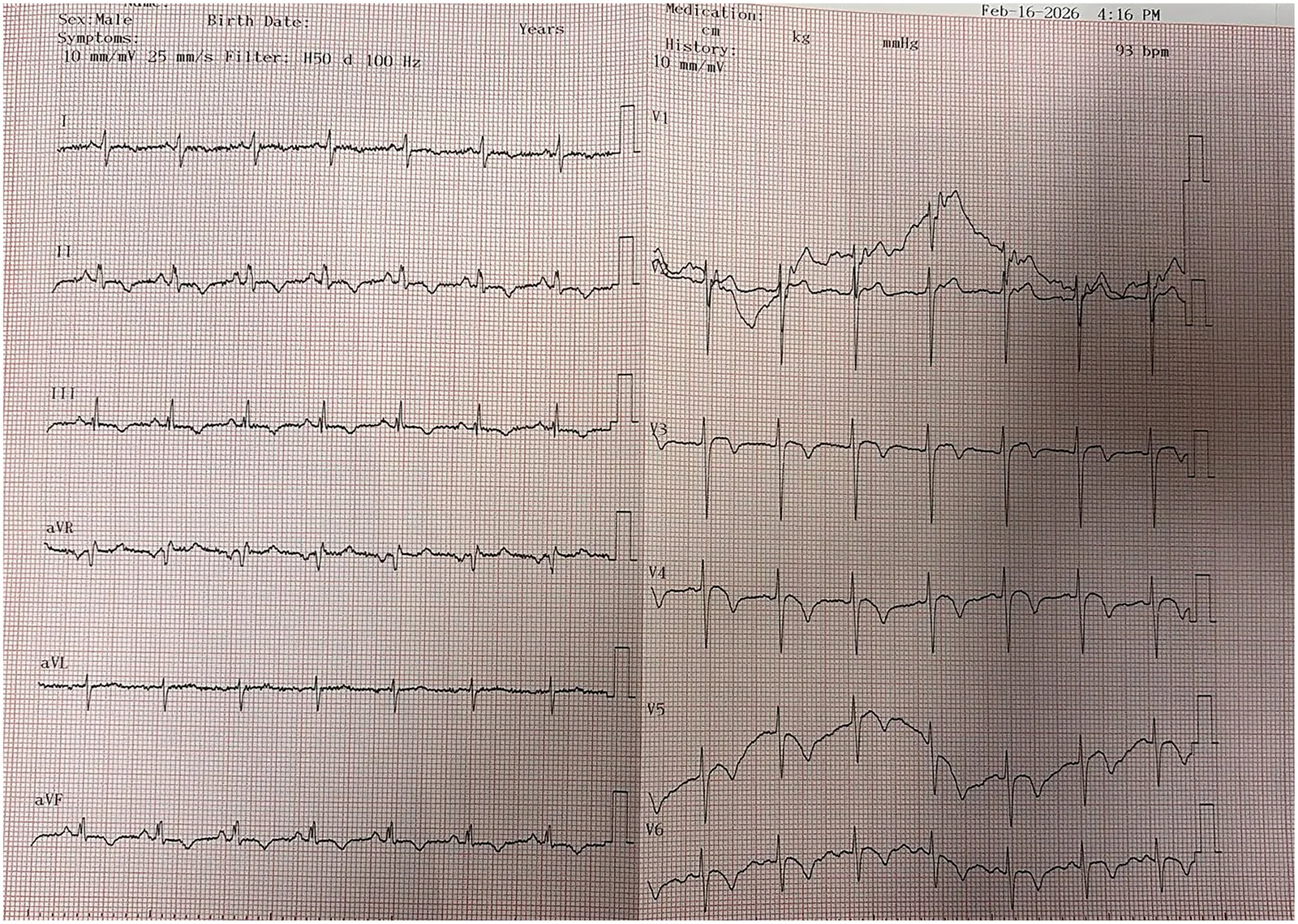
The patient's ECG following medical cardioversion.

**Figure 3:**
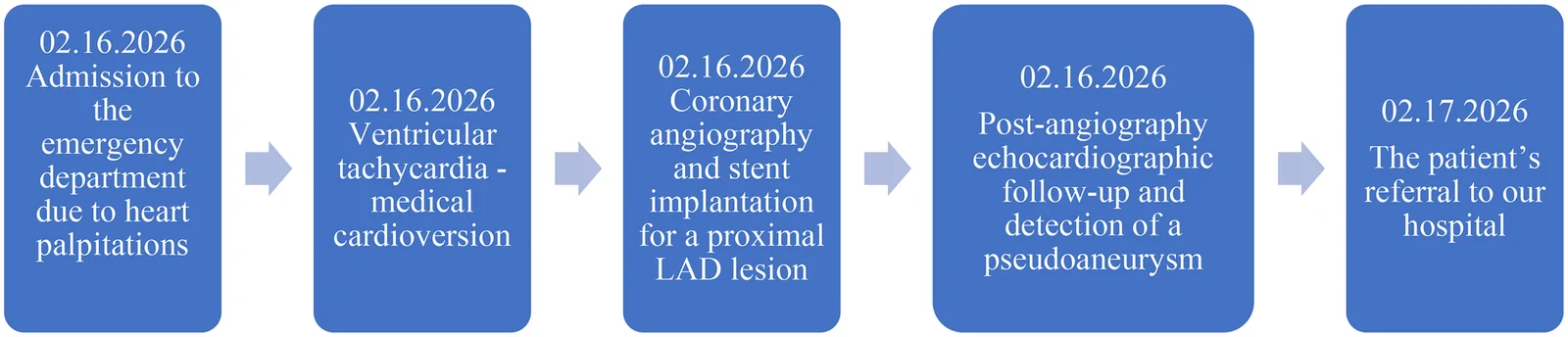
The patient's process prior to referral to our hospital.

A 38-year-old male patient with no known medical history presented to the emergency department while in prison, with complaints of palpitations during a football match. An electrocardiogram (ECG) revealed ventricular tachycardia; sinus rhythm was restored after intravenous administration of 300 mg of amiodarone (Figure 1). A patient with no history of chest pain but with T-wave negativity in the anterior and inferior leads on a follow-up ECG and an elevated troponin-I level of 96 ng/L and 143.10 ng/L (normal range 0–25 ng/L)(The first is upon admission to the hospital; the second troponin level is measured 1 h later) underwent coronary angiography (Figure 2). A patient with a thrombosed lesion in the proximal left anterior descending artery (LAD) on coronary angiography underwent percutaneous coronary angioplasty (Supplementary Video S1). Following the patient's loading doses of ticagrelor and acetylsalicylic acid, the daily doses were as follows: ticagrelor 90 mg twice daily, acetylsalicylic acid 100 mg once daily, metoprolol 50 mg once daily, ramipril 2.5 mg once daily, furosemide 40 mg IV once daily, enoxaparin 2 doses of 6,000 IU subcutaneously, and cefazolin 3 doses of 1 g IV. An echocardiogram was performed on the patient during follow-up, as one had not been performed prior to the angiography. On the follow-up echocardiogram, hypokinesis was observed in the anterior wall and septum, along with an appearance consistent with a thrombus-filled pseudoaneurysm at the apex; the ejection fraction (EF) was 30%. The patient was referred to us for detailed examination and evaluation (Figure 3).

**Figure 4:**
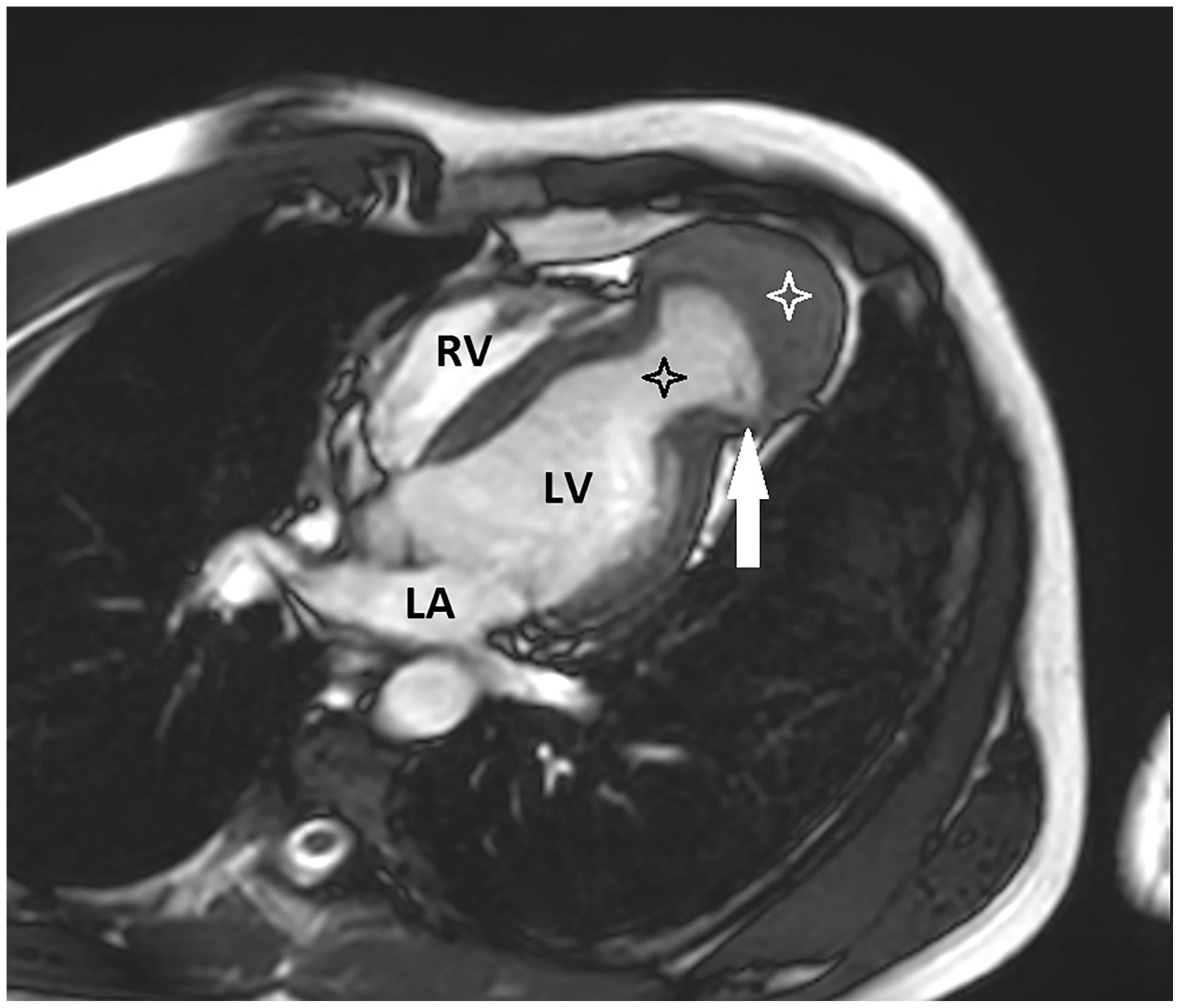
Patient's cardiac MRI image showing a left ventricular pseudoaneurysm(4-chamber cine, horizontal long-axis).

A cardiac magnetic resonance imaging (MRI) scan was performed. The MRI revealed systolic dysfunction in the left ventricle. Akinetic segments were observed in the basal-anterior, anteroseptal, and mid-inferolateral walls, along with wall thinning and hypokinesis in other segments. Additionally, in the akinetic segments, subendocardial contrast enhancement in an ischemic pattern was observed on late-phase contrast-enhanced PSIR images. A defect was noted in the LV apical wall, representing a pseudoaneurysm measuring 55 × 50 × 46 mm that extended into the pericardial space, with a partially thrombosed lumen, an inflamed surrounding area, and a thick pericardium covering it (Figure 4). A patient who had not undergone echocardiography prior to angiography and in whom a pseudoaneurysm was detected following angiography was evaluated for percutaneous closure and surgical intervention. Given that our clinic has experience only with percutaneous closure of the ventricular septum and that the patient's MRI revealed a pseudoaneurysm in the apical region of the left ventricle, we decided to proceed with surgery rather than a percutaneous procedure. The patient was referred to the cardiovascular surgery department.

**Figure 5:**
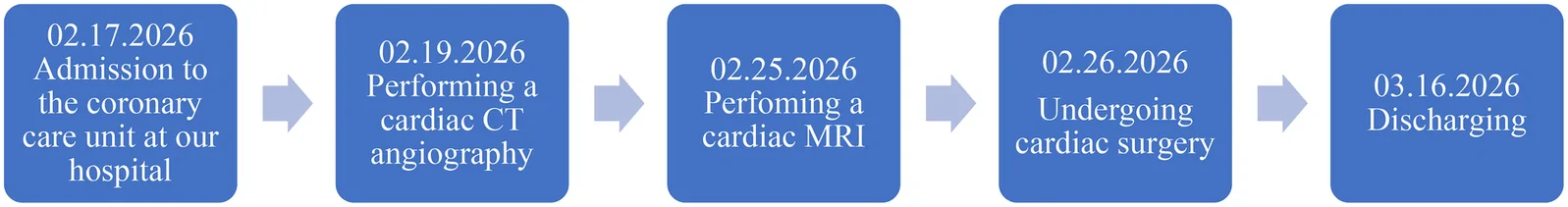
Process workflow chart for procedures performed at our hospital.

The pseudoaneurysm sac was surgically opened, the thrombus material inside was removed, and the ventricular defect was repaired with a biological patch (Supplementary Figures S1, S2). Following the operation, the patient was switched from ticagrelor to clopidogrel. The patient was hospitalized for 18 days postoperatively. Following a postoperative echocardiogram showing no evidence of a pseudoaneurysm or thrombus, the patient was discharged with a medical treatment plan (Figure 5). When discharged, the patient was prescribed clopidogrel 75 mg once daily, acetylsalicylic acid 100 mg once daily, ramipril 2.5 mg once daily, metoprolol 50 mg tablet once daily, ciprofloxacin 750 mg twice daily, and amoxicillin+clavulanic acid 1,000 mg twice daily.

At the 3-month postoperative follow-up, the patient had no limitations in functional capacity. The echocardiogram showed an EF of 42%, and no evidence of a thrombus in the left ventricle.

## Dıscussıon

A ventricular pseudoaneurysm is a rare complication of acute myocardial infarction, cardiac trauma, or cardiac surgery that can be life-threatening if not treated immediately (4). Patients may present with symptoms such as chest pain, shortness of breath, palpitations, or arrhythmia, or they may be diagnosed during an imaging examination even if they are completely asymptomatic (5, 6). Transthoracic echocardiography, transesophageal echocardiography, cardiac computed tomography, and cardiac MRI are used to diagnose ventricular pseudoaneurysms (4).

Due to the 30%–45% risk of rupture, ventricular pseudoaneurysms are typically treated surgically; however, in patients for whom surgery is too high-risk, percutaneous closure may be performed (4, 7, 8).

In the present case, the patient presented with ventricular tachycardia, and subsequent echocardiographic evaluation revealed a ventricular pseudoaneurysm.

Since ventricular tachycardia can be the first sign of acute myocarditis, this condition was considered in the differential diagnosis. However, the thrombotic lesion in the proximal left anterior descending (LAD) artery detected on coronary angiography, along with the subendocardial ischemic contrast enhancement pattern observed on cardiac MRI, strongly ruled out myocarditis, which typically presents with non-ischemic epicardial or mid-wall contrast enhancement patterns. In addition to myocarditis, coronary embolism and spontaneous coronary artery dissection were also considered. However, the angiographic findings did not suggest coronary embolism or SCAD, and no imaging findings were identified to support these etiologies.

Despite the fact that acute ischemia contributed to the initial ventricular tachycardia, the presence of a large left ventricular pseudoaneurysm and extensive myocardial scar tissue may have provided a significant substrate for scar-related re-entry. However, because VT occurred before definitive imaging was performed, the exact extent to which ischemia and the pseudoaneurysm contributed to the arrhythmia cannot be definitively determined. It is highly likely that both mechanisms contributed to the arrhythmic episode.

If an echocardiogram had been performed prior to the angiography, the patient would have been referred for surgery without proceeding with percutaneous coronary intervention. Due to our inexperience with apical percutaneous closure, we opted for surgical treatment for the patient's safety and survival. The patient successfully underwent surgical repair.

## Conclusıon

Ventricular pseudoaneurysm is a rare but potentially fatal mechanical complication that may present with arrhythmias, including ventricular tachycardia. Cardiac imaging techniques play a crucial role in diagnosis and treatment decisions. Early diagnosis and immediate treatment are vital for preventing serious complications such as rupture and sudden cardiac death.

## Data Availability

The raw data supporting the conclusions of this article will be made available by the authors, without undue reservation.

## Supplementary material

Supplementary Figure S1 LV pseudoaneurysm during surgery.

Supplementary Figure S2 Thrombus within the LV pseudoaneurysm sac.

Supplementary Video S1 Angiographic view of the patient's LAD lesion.
